# Implementing Explainable Machine Learning Models for Practical Prediction of Early Neonatal Hypoglycemia

**DOI:** 10.3390/diagnostics14141571

**Published:** 2024-07-19

**Authors:** Lin-Yu Wang, Lin-Yen Wang, Mei-I Sung, I-Chun Lin, Chung-Feng Liu, Chia-Jung Chen

**Affiliations:** 1 Department of Pediatrics, Chi Mei Medical Center, Tainan City 71004, Taiwan; linyu870203@gmail.com (L.-Y.W.); yen3546@yahoo.com.tw (L.-Y.W.); rstro_boy@yahoo.com.tw (I.-C.L.); 2 Center for General Education, Southern Taiwan University of Science and Technology, Tainan City 71005, Taiwan; 3 Department of Medicine, College of Medicine, Kaohsiung Medical University, Kaohsiung City 81201, Taiwan; 4 Department of Childhood Education and Nursery, Chia Nan University of Pharmacy and Science, Tainan City 71710, Taiwan; 5 Department of Medical Research, Chi Mei Medical Center, Tainan City 71004, Taiwan; mayyi323@gmail.com; 6 Department of Information Systems, Chi Mei Medical Center, Tainan City 71004, Taiwan; carolchen@mail.chimei.org.tw

**Keywords:** neonatal hypoglycemia, term, late preterm, prediction model, machine learning, explainability, hospital information system

## Abstract

Hypoglycemia is a common metabolic disorder that occurs in the neonatal period. Early identification of neonates at risk of developing hypoglycemia can optimize therapeutic strategies in neonatal care. This study aims to develop a machine learning model and implement a predictive application to assist clinicians in accurately predicting the risk of neonatal hypoglycemia within four hours after birth. Our retrospective study analyzed data from neonates born ≥35 weeks gestational age and admitted to the well-baby nursery between 1 January 2011 and 31 August 2021. We collected electronic medical records of 2687 neonates from a tertiary medical center in Southern Taiwan. Using 12 clinically relevant features, we evaluated nine machine learning approaches to build the predictive models. We selected the models with the highest area under the receiver operating characteristic curve (AUC) for integration into our hospital information system (HIS). The top three AUC values for the early neonatal hypoglycemia prediction models were 0.739 for Stacking, 0.732 for Random Forest and 0.732 for Voting. Random Forest is considered the best model because it has a relatively high AUC and shows no significant overfitting (accuracy of 0.658, sensitivity of 0.682, specificity of 0.649, F1 score of 0.517 and precision of 0.417). The best model was incorporated in the web-based application integrated into the hospital information system. Shapley Additive Explanation (SHAP) values indicated mode of delivery, gestational age, multiparity, respiratory distress, and birth weight < 2500 gm as the top five predictors of neonatal hypoglycemia. The implementation of our machine learning model provides an effective tool that assists clinicians in accurately identifying at-risk neonates for early neonatal hypoglycemia, thereby allowing timely interventions and treatments.

## 1. Introduction

Hypoglycemia refers to low blood sugar levels and is one of the common metabolic disorders in the neonatal period. The prevalence of hypoglycemia in full-term newborns varies from 3% to 29% between studies [1,2,3,4,5]. During intrauterine life, the mother continues to supply glucose to the fetus through the placenta; however, after birth, the glucose supply from the mother abruptly stops. As a result, the newborn’s blood sugar levels may significantly decrease, sometimes dropping to 20–25 mg/dL within 1–2 h after birth [4,6]. Various reports recommended routine blood glucose screening for all-term newborns with a body weight below the 10th percentile [7,8]. Our recent data [9] indicate that 19.4% of small-for-gestational-age (SGA) infants developed hypoglycemia and approximately 97% of early hypoglycemic episodes occur within the first 2 h after birth. Newborns with hypoglycemia may experience poor feeding, tremors, hypothermia, lethargy, seizures, apnea, tachypnea, and cyanosis [10,11,12]. Prolonged and untreated hypoglycemia can cause brain injury and adverse neurodevelopmental outcomes such as learning disabilities and mental retardation [1,13,14]. Therefore, early identification of neonates at risk of developing hypoglycemia and prompt management are crucial aspects of newborn care, as they can reduce mortality and morbidity induced by hypoglycemia.

According to the clinical guidelines published by the American Academy of Pediatrics (AAP) [6], universal screening for hypoglycemia is recommended for high-risk neonates. High-risk neonates include those who are late preterm, small for gestational age (SGA), with intrauterine growth restriction, large for gestational age, and infants of diabetic mothers. They recommend frequent measurement of glucose concentrations one hour after birth and then before feeds every 3–4 h for the first 24 h of life; they also added screening every 3–8 h for the second 24 h of life. In Taiwan, newborns typically undergo a 4 h observation period in the well-baby nursery after admission. According to Taiwan’s well-baby newborn nursery policy, glucose levels in the first 4 h in at-risk infants must be routinely checked. Therefore, all high-risk neonate’s blood glucose levels are checked in the first 30 min and then at 1 h, 2 h, and 4 h of age to monitor neonatal hypoglycemia within the first 4 h of birth based on the AAP recommendation in the well-baby nursery.

However, frequent blood glucose screening for diagnosing neonatal hypoglycemia can cause stress for parents and disrupt the interaction between newborns and their caregivers. Moreover, repeated heel stick punctures, a painful procedure associated with blood glucose testing, can lead to increased nursing care time and potential complications such as skin lacerations, heel bone osteomyelitis, hematoma, and skin necrosis [15].

In recent years, the application of artificial intelligence (AI) and machine learning (ML) in medical and healthcare systems has rapidly expanded [16,17]. With the growth and accessibility of health data, ML is widely employed to analyze large-scale medical data, aiding clinical decision-making, enhancing healthcare quality, improving efficiency, and reducing costs [18,19,20]. These tasks include image detection [21], outcome prediction, risk analysis [22], lifestyle management [23], readmission risk assessment [24], COPD diagnosis [25], malaria diagnosis [26], and COVID-19 detection, which played a pivotal role in pandemic control [27,28]. In the field of neonatology, AI/ML also demonstrates its benefits for clinical decision-making, such as predicting the risk of death in neonates and infants [29,30], and improving the quality of care in the Neonatal Intensive Care Unit (NICU) by predicting various outcomes such as extubation results, readmissions, length of stay, mortality [31], neonatal sepsis [32], severe morbidity [33], and apnea among premature neonates [34]. The use of AI/ML for hypoglycemia prediction has also increased, though studies specifically predicting neonatal hypoglycemia shortly after birth remain limited.

In this study, we aim to develop a predictive model for neonatal hypoglycemia using electronic medical records from fewer and routine administrative data. The emergence of AI/ML technology presents promising opportunities for advancing this endeavor. Our ML model is designed to swiftly and accurately identify newborns at risk of hypoglycemia in well-baby nurseries, providing clinicians with valuable insights. More importantly, we implemented a web-based AI prediction application, incorporating the best-performing machine learning model developed in this study, which is currently operational in clinical settings.

This study is expected to make significant contributions to both academia and practice. Our innovative approach extends the methodologies in predicting neonatal risk, and the implementation of the proposed model has the potential to reduce the frequency of blood glucose testing. This reduction could lead to fewer heel stick punctures, thereby improving patient comfort, enhancing nursing efficiency, and ultimately lowering healthcare costs.

## 2. Materials and Methods

### 2.1. Study Design and Setting

**Figure 1:**
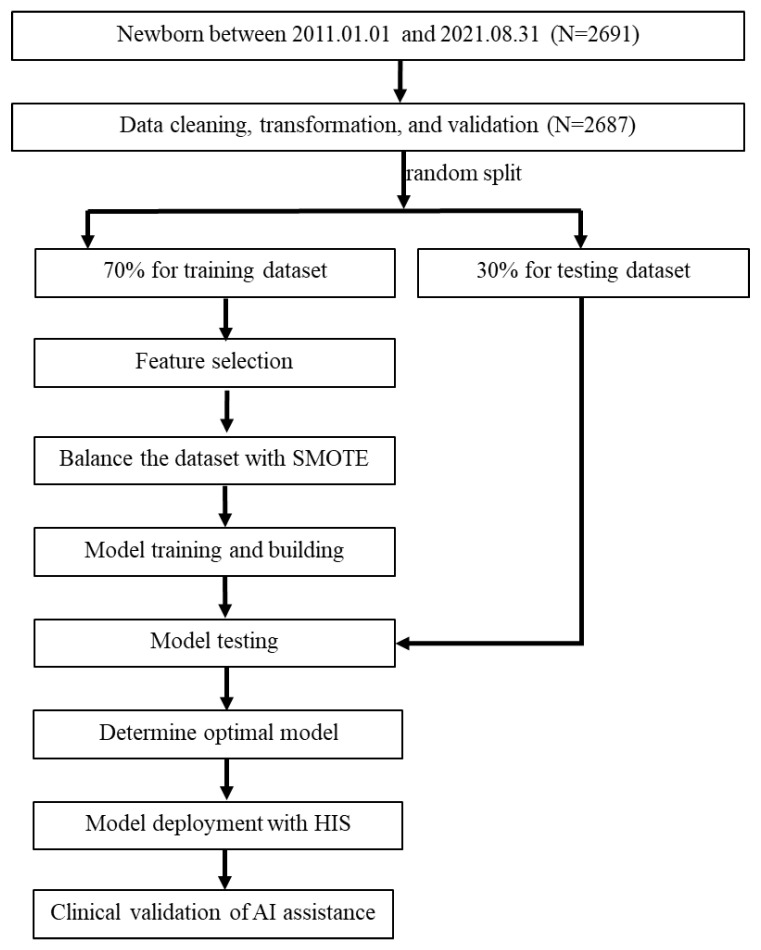
Flow chart of the current study. SMOTE, synthetic minority oversampling technique; HIS, hospital information system.

The present study formed a multidisciplinary team comprising domain experts (pediatric physicians and nurse practitioners), data scientists, and information technology engineers. We retrospectively collected the electronic medical records of newborns (gestational age > 35 weeks) admitted to the well-baby nursery between 1 January 2011 and 31 August 2021 who underwent blood glucose concentration monitoring according to the nursery protocol. We obtained approval from the Institutional Review Board of the hospital before data collection (IRB No.: 11203-001). Newborns included in the study were those who were either small or large for their gestational age, born to diabetic mothers, or those who clinically exhibited symptoms suggestive of hypoglycemia. We excluded infants with perinatal asphyxia, congenital malformations, shock, and endocrine deficits, as well as patients with missing items in their medical records. Data from 2691 newborns were primarily included, but 4 newborns were excluded due to missing items (mother’s height) in their medical records. Therefore, 2687 newborns were included in the predictive model analysis, as illustrated in the study flow chart (Figure 1).

Initially, we identified 24 factors through a literature review and expert input, extracted from the medical records of the infants and their mothers as potential feature variables. The cutoff value for blood glucose level was set at ≤45 mg/dL for establishing prediction models. Subsequently, the feature selection process was conducted to determine the final selected features for modeling with machine learning approaches.

Figure 1 illustrates the flow chart for integrating AI/ML into the prediction model for hypoglycemia in the well-baby nursery. Initially, 2691 newborns were included in the study. Subsequently, data extraction, transformation, and validation were performed from the hospital information system (HIS) into a data mart, with meticulous handling of missing and incomplete data. Since only four cases had missing data (a very small proportion), and none of them involved hypoglycemia, these cases were directly deleted. Some continuous variables were transformed into categorical variables using a one-hot encoding approach for model training purposes. For example, sex was transformed into a binary category and infant weight was transformed into two binary variables (BW < 2500 gm and BW > 4000 gm). The entire dataset was manually validated by two researchers with a medical background. As a result, a total of 2687 neonates were included in the prediction model analysis. Next, potential feature variables were selected for model training, and multiple models were developed for comparison. The 2687 neonates were randomly divided into a training dataset (70%, *n* = 1880) and a testing dataset (30%, *n* = 807) to develop prediction models. This division ensured the independence of the test dataset from the training dataset. To address imbalanced outcome samples, the synthetic minority oversampling technique (SMOTE) [19,35,36,37] was employed to balance the minority cases in the training dataset, while the testing dataset was kept without any oversampling. The number of positive outcomes in the training dataset before SMOTE processing was 506, and after SMOTE processing, it increased to 1374, equal to the number of negative outcomes.

Nine machine learning methods were used for modeling early neonatal hypoglycemia prediction. The best-performing model was selected based on AUC and the presence of overfitting. Finally, the best model was deployed in the AI web service and integrated into the HIS in the well-baby nursery. Following six months of pilot testing and validation, the application was launched in the HIS to aid physicians in real-time decision-making. After a 2-month promotional period following the launch of the prediction application, we collected data on newborns to compare the AI predictions with the actual occurrences of hypoglycemia.

### 2.2. Feature Selection and Model Building

The raw dataset consisted of 24 feature variables including maternal and neonatal clinical data collected post-birth. Feature selection was guided by a literature review and consensus among our multidisciplinary team of pediatric experts. Additionally, variables were also considered based on their relevance to characterizing neonatal hypoglycemia, routine availability, and ease of interpretation with clear clinical significance.

We used Spearman correlation analysis to help with feature selection by examining statistically significant clinical variables, results of Spearman’s correlation coefficient, and experts’ opinions [38] were employed for the final feature decision. To prevent data leakage in validation, only the training dataset underwent univariate significance testing using a *T*-test or chi-square test at a significance level of *p* < 0.05 (using SPSS software, version 19.0, IBM Corp., Chicago, IL, USA). We first screened the variables that exhibited statistical significance (*p* < 0.05) but excluded ones with very low correlation (we set a coefficient threshold of 0.05); we also included some variables based on expert clinical experience though they were not statistically significant. Subsequently, the study built models using nine machine learning methods, including Logistic regression, Random Forest, XGBoost, LightGBM, Multilayer Perceptron (MLP), Support Vector Machine (SVM), AdaBoost, Stacking and Voting to predict neonatal hypoglycemia. The cutoff values of blood glucose level ≤ 45 mg/dL were selected as the positive outcome for establishing prediction models.

Logistic regression is a statistical approach and can be regarded as a supervised ML algorithm developed for classification problems. SVM handles regression, binary, and multi-class classification by finding a hyperplane that maximizes the distance between classes. MLP is a feedforward artificial neural network model that maps sets of input data onto a set of appropriate outputs.

Ensemble methods [39] are advanced machine learning techniques that combine multiple models to enhance prediction performance and stability, reducing the risk of overfitting such as Random Forest, XGBoost, LightGBM, AdaBoost, Stacking and Voting. Random Forest contains a multitude of decision trees and are generated from a number of combined optimization decision trees. Random Forest is a fast and efficient ensemble ML method for classification and regression. XGBoost is a powerful gradient boosting framework known for its speed and performance. It uses a level-wise tree growth algorithm, which helps control overfitting. LightGBM is also a highly efficient gradient boosting framework designed for speed and low memory usage. It uses a leaf-wise tree growth algorithm, which can result in faster training and better performance on large-scale and high-dimensional data. Adaptive Boosting (AdaBoost) improves prediction by combining multiple weak classifiers. It trains classifiers sequentially, each focusing on samples misclassified by the previous ones. Misclassified samples have higher weights in subsequent rounds. The final prediction is a weighted vote of all classifiers, enhancing overall accuracy.

The Voting algorithm uses methods like majority voting, where each model’s prediction is counted as a vote, where predictions are weighted by confidence scores. The Stacking algorithm leverages the complementarity among base models to enhance performance and generalization. It consists of two phases: base model training and meta-model training. The meta-model is trained on this new dataset, and its performance is evaluated on the combined predictions from the testing sets of the base models. In this study, the stacking approach employed Logistic Regression, Random Forest, XGBoost, LightGBM, MLP, SVM, and AdaBoost as base models, with a Logistic Regression classifier serving as the meta-model.

### 2.3. Model Performance Measurement

This study utilized accuracy, sensitivity, specificity, AUC-ROC (area under the receiver operating characteristic curve), precision, and F1 score as metrics to evaluate the performance of the predictive model. These metrics have been widely employed in healthcare research [36,40] and machine learning modeling [19,25]. Accuracy represents the proportion of true results, either true positive or true negative, in the targeted population. It measures the degree of veracity of a diagnostic test on a condition. Sensitivity represents the proportion of true positives that are correctly identified by a diagnostic test. It demonstrates how good the test is at detecting hypoglycemia in neonates in the well-baby nursery. Specificity represents the proportion of the true negatives correctly identified by a diagnostic test. It indicates how good the test is at identifying a normal (negative) condition. An ROC curve (receiver operating characteristic curve) is a graphical representation of a classification model’s performance across all classification thresholds, while AUC (area under the curve) quantifies the overall separability of the model. AUC-ROC (area under the receiver operating characteristic curve) was employed to identify the best model, where an AUC value of 1 signifies a perfect test and 0.5 indicates a worthless test [41,42]. The model with the highest AUROC was selected to develop a computer application that was later integrated into the computerized physician ordering entry system and well-baby nursery system.

Shapley Additive Explanation (SHAP) values are based on game theory and explain the accurate attribution of each feature in a model [43]. Features with positive SHAP values positively influence the prediction, whereas those with negative values exert a negative impact; the magnitude indicates the strength of the effect. The SHAP values were employed in this study to interpret, which feature variables were most important in the best prediction model. All *p* values were two-sided, and a value of less than 0.05 was considered significant.

### 2.4. Model Deployment with HIS

To demonstrate the usability and acceptance of our AI models, AI risk prediction applications using the best model were developed. These prediction applications, for both individual predictions and predicting all newborns (via a dashboard), were integrated into the HIS to provide clinical assistance for healthcare professionals. The models were constructed using the Python programming language (version 3.7.0) using the scikit-learn package, while the web-based interface was developed in MS Visual Studio^®^ utilizing VB (version 17.7).

### 2.5. Clinical Validation of AI Assistance

After the prediction application was launched, we collected data on hypoglycemia occurrences in newborns between July 2023 and December 2023 to validate the clinical efficacy of the AI application.

## 3. Results

### 3.1. Demographics of the Hypoglycemic and Non-Hypoglycemic Groups

A total of 2687 high-risk neonates were recruited for this study. Among them, 26.9% (723) were diagnosed with hypoglycemia, while 73.1% (*n* = 1964) had euglycemia. The neonates were randomly divided into a training dataset (70%, *n* = 1880) and a testing dataset (30%, *n* = 807) to develop prediction models. For model feature selection to prevent data leakage in validation, only the training dataset underwent univariate significance testing (Table 1).

Table 1 presents the demographic and significant variable of neonates, comparing those with and without hypoglycemia within the first 4 h of birth in the training dataset. Of the 506 hypoglycemic neonates, 252 (49.8%) are males and 254 (50.2%) are females.

As shown in Table 1, neonates in the hypoglycemia group were those with low gestational age (*p* < 0.001), body weight below 2500 gm (*p* < 0.001) or with a low Apgar score at 1 min (*p* < 0.005). Neonates born via cesarean delivery accounted for the majority of the hypoglycemia cases, comprising 61.86% of them (*n* = 313, *p* < 0.001). Furthermore, hypoglycemia was more prevalent among neonates with respiratory distress (*n* = 157, *p* < 0.001), and Polycythemia (*n* = 17, *p* < 0.001).

### 3.2. Correlation between Feature Variables and Hypoglycemia

**Figure A1:**
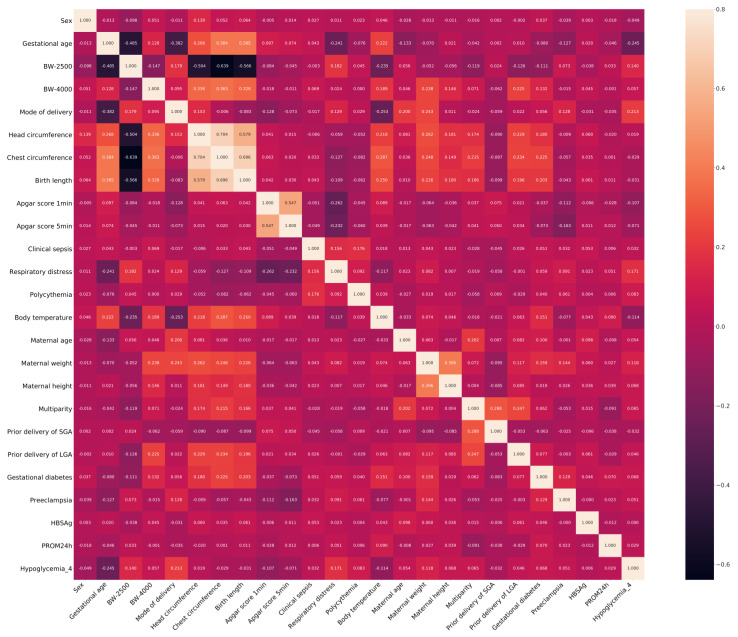
Heatmap of the correlation.

To efficiently select appropriate features for machine learning, Spearman’s correlation coefficient analysis was conducted between feature variables and neonatal hypoglycemia (Table 2). Based on the analysis, the 24 feature variables were ranked according to their correlation (absolute value of correlation coefficient) to neonatal hypoglycemia, the top five correlations are gestational age, mode of delivery, respiratory distress, BW-2500, and maternal weight. A heatmap displaying Spearman’s correlation coefficients is presented in Figure A1.

### 3.3. The Selected Features

Based on the feature selection procedure mentioned above, we initially identified 12 variables that were statistically significant or close to significance (*p* ≤ 0.055). Polycythemia, although significant, was excluded due to the small number of cases and the difficulty in obtaining it right after the baby is born. Maternal height and weight were combined into the BMI (body mass index) variable. Additionally, clinical experience and the literature suggested that multiparity and higher birth weight (>4000 gm) were associated with an increased risk of neonatal hypoglycemia, leading us to include these variables. Therefore, a total of 12 feature variables were ultimately selected for modeling. This included gestational age, birth weight < 2500 gm, birth weight > 4000 gm, mode of delivery, Apgar score at 1 min, clinical sepsis, respiratory distress, hypothermia (body temperature), BMI, multiparity, gestational diabetes mellitus, and preeclampsia.

### 3.4. The Predictive Models Using the 12 Feature Variables

This study employed 9 machine learning algorithms (Logistic Regression, Random Forest, Light GBM, XG Boost, SVM, AdaBoost, MLP, Stacking, Voting) using 12 feature variables to build predictive models for early neonatal hypoglycemia. Grid search with 5-fold cross-validation for hyper-parameters (Table A1) tuning for each algorithm was conducted to obtain an optimal model.

**Figure A2:**
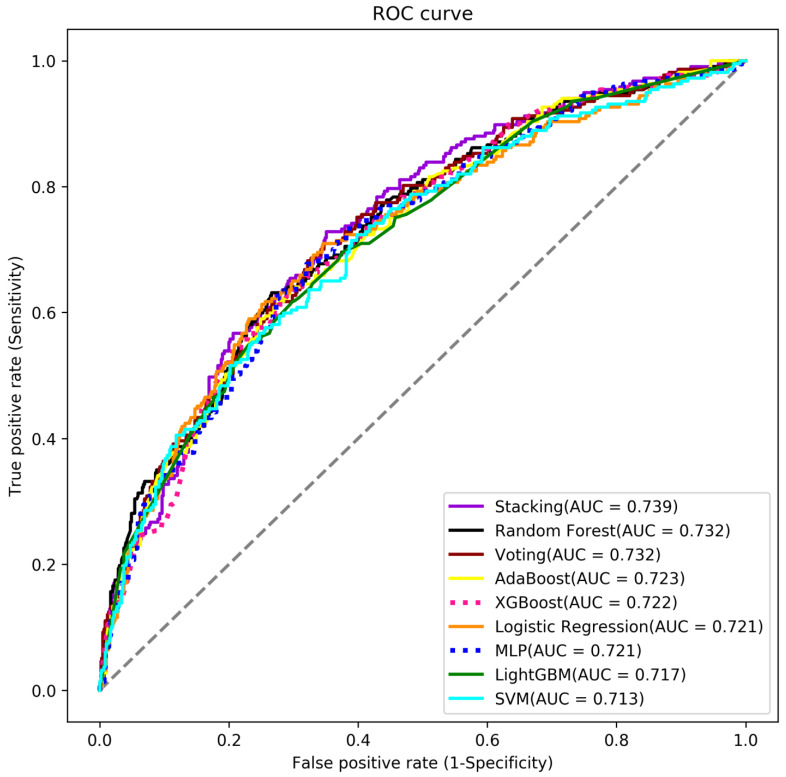
The ROC curve for all models.

**Figure A3:**
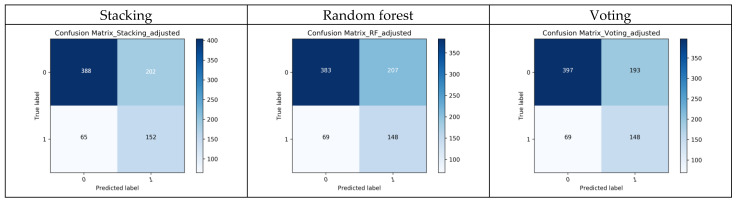

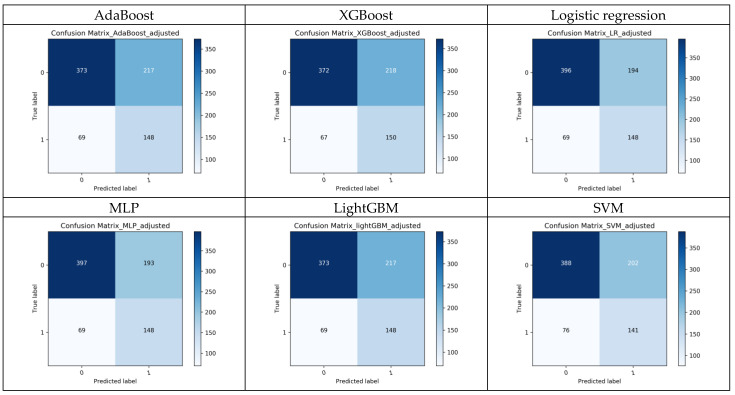
The confusion matrices of the 9 models.

Table 3 summarizes the performance of these models. The primary criterion for model selection in this context is the AUC value, as it provides a comprehensive measure of the model’s ability to distinguish between classes. A higher AUC indicates better overall performance. We found that the Stacking model has the highest AUC of 0.739, followed by the Random Forest model with 0.732 and the Voting model with 0.732. The ROC curves for all models are shown in Figure A2. The confusion matrices of each model were summarized in Figure A3.

**Figure A4:**
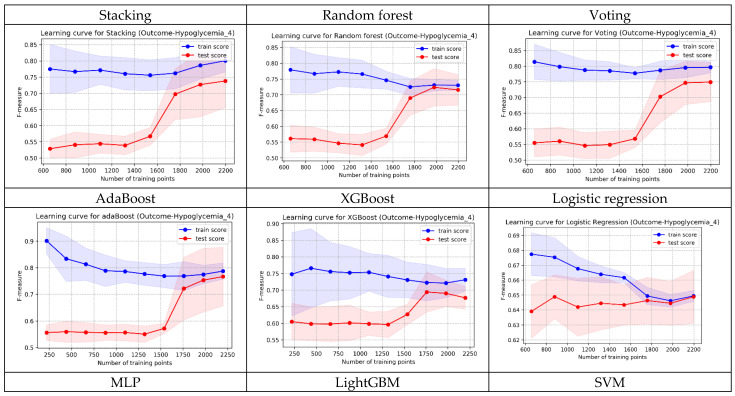

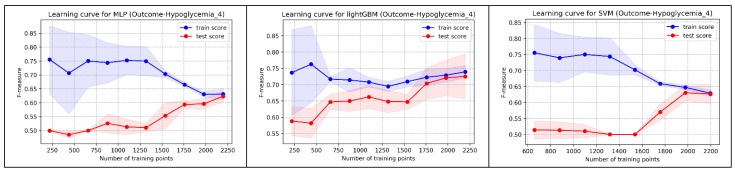
The shapes of learning curves of the 9 models.

In addition to AUC, we also evaluated the learning curve of each model. While the AUC of Random Forest was slightly lower than Stacking, its convergence in the shape of learning curves (training and testing) was superior to both Stacking and Voting. Following multidisciplinary team discussion, Random Forest was selected as the best prediction model for implementation in the well-baby nursery. Figure A4 displays the learning curve shapes of each model.

### 3.5. Explainability of the Best Prediction Model

**Figure 2:**
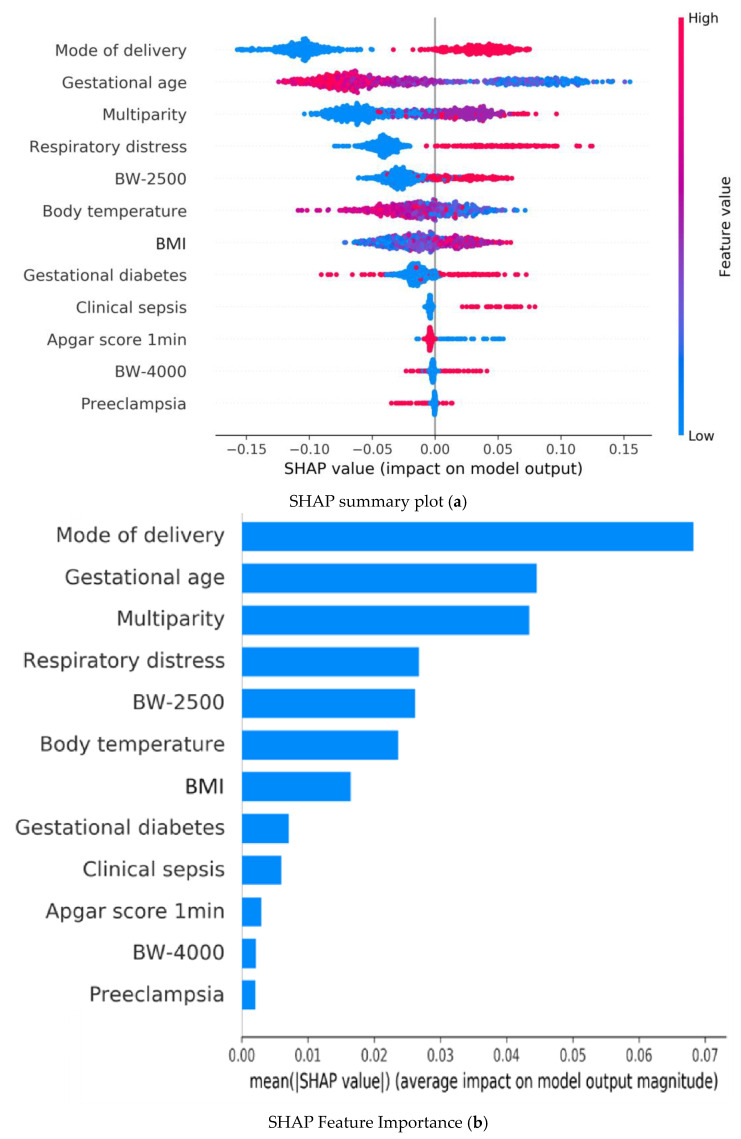
Random forest model explainability with SHAP: (**a**) Summary plots of Shapley Additive Explanations (SHAP) in the Random Forest model illustrating the influence of each input variable on predicting hypoglycemia; red and blue indicate higher and lower input values, respectively. A red plot in the positive range of SHAP values indicates a higher input value corresponding to a higher Contribution Level (CL) value, affecting the output prediction. (**b**) Summary bar lot of Shapley Additive Explanations (SHAP) in the Random Forest model illustrating the mean value of absolute SHAP values for each input variable within the Random Forest model, highlighting the average impact of each feature on the prediction outcomes. BW-2500: BW < 2500 gm; BW-4000: BW < 4000 gm.

Figure 2 illustrates the SHAP summary plot of the Random Forest model using the 12 features to identify the most important ones that influenced the prediction model. A feature with a higher SHAP value indicates a higher probability of hypoglycemia based on the prediction model. In Figure 2a, colored plots represent SHAP values. The red and blue plots in the SHAP indicate larger and smaller values, respectively, which suggest that increasing or decreasing values will correspondingly increase or decrease the predicted probability of hypoglycemia.

Bar plots in Figure 2b represent the mean of the absolute SHAP value (MEAN[SHAP]) for each feature variable. It can be seen that the top five important feature variables in descending order are as follows: cesarean section, gestational age, respiratory distress, multiparity, and hypothermia.

### 3.6. Computer-Assisted Prediction Application Development

To evaluate the usability of the constructed machine learning models, an AI risk prediction system for clinical use was developed using the best-performing model. This system was implemented in the nursery to assist physicians and nurses in real-time monitoring of neonatal hypoglycemia.

**Figure 3:**
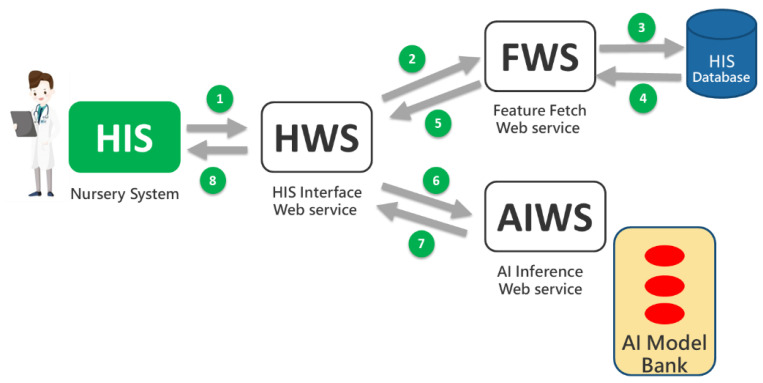
The AI web service framework.

Based on Tsai’s AI web service framework [44], three types of web services were developed: HWS (HIS Interface Web Service), FWS (Feature Retrieval Web Service), and AIWS (AI Computation Web Service). These services support the implementation of individual prediction functions and the digital dashboard, as illustrated in Figure 3. When users (e.g., physicians and nurses) initiate a prediction through the HIS (nursery system), eight messages are exchanged between the web services following the steps numbered 1 through 8 in Figure 3. The prediction models were developed using the Python programming language, while the web service software was developed using MS Visual Studio 2019 with Visual Basic^®^.

The HWS receives prediction requests from the HIS and forwards them to the FWS to retrieve necessary feature values such as maternal BMI and gestational age. These values are then sent to the AIWS, which uses the AI model from Chi Mei’s model bank to generate a prediction. The AIWS performs the prediction and sends the result back to the HWS, which then returns the prediction result to the HIS for display to users (physicians and nurses).

**Figure 4:**
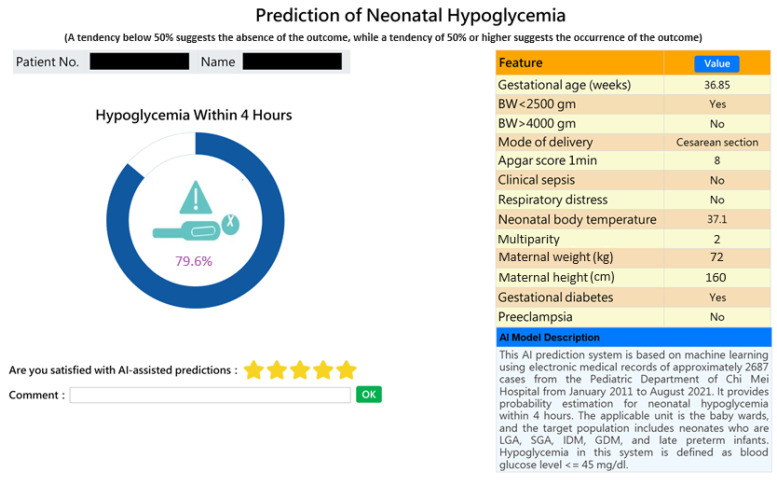
Snapshot of an AI-powered web application predicting early neonatal hypoglycemia. The application provides individual predictions for early neonatal hypoglycemia with detailed feature values.

This AI-based web prediction application was integrated into the existing HIS, specifically the nursery system and inpatient physician ordering system, to assist physicians and nurses in making informed decisions regarding the care of high-risk neonates and in communicating with parents. A probability of greater than or equal to 50% indicates a tendency for the risk to occur, while less than 50% indicates a tendency for the risk not to occur—the higher the probability, the higher the risk. Figure 4 provides a snapshot of the web service application for individual prediction of a high-risk case.

**Figure 5:**
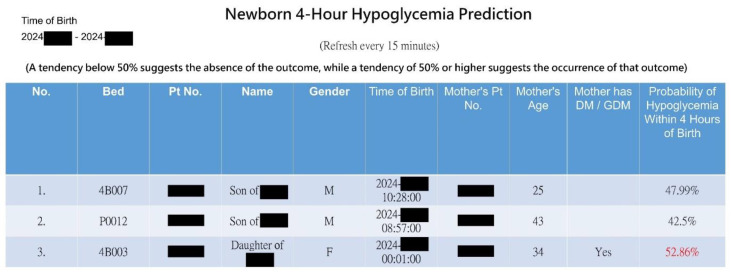
Snapshot of the AI web dashboard predicting hypoglycemia risk for all newborns in the nursery room.

With innovation, we expanded individual predictions (Figure 4) into a web-based dashboard for real-time monitoring of all infants in the nursery room at a glance (see Figure 5). Upon the admission or discharge of a baby to the nursery room, the dashboard automatically updates the prediction results every 15 min for all babies present. The webpage presents a graphical representation of the risk probabilities of neonatal hypoglycemia within 4 h, highlighting high-risk infants in red. This enables healthcare members to quickly identify high-risk infants, facilitating timely intervention to prevent hypoglycemia.

### 3.7. Result of Clinical Validation of AI Assistance

After a 2-month promotional period following the launch of the prediction application, we collected data on 152 newborns (referred to as the hold-out set) to compare the AI predictions with the actual occurrences of hypoglycemia. Among these newborns, eight experienced hypoglycemia within the first four hours. The AI predicted probabilities for these eight newborns ranged from 0.512 to 0.594, demonstrating excellent predictive accuracy of 70.4%, as well as sensitivity of 100% and specificity of 68.8%. In total, 99 newborns without hypoglycemia displayed probabilities below 0.5. Consequently, it may be advisable that if the predicted probability is below the threshold of 0.5, exemptions from glucose blood needle sticks or a reduction in their frequency (e.g., needle sticks every two hours) could be considered. Based on this approach and following the AAP’s recommendations, we anticipate a most optimistic potential reduction of 396 blood glucose tests (99 × 4). However, close observation of the baby for signs of hypoglycemia (such as drowsiness, refusal to feed, abnormal crying, etc.) should still adhere to standard procedures. This approach could reduce the risk of blood needle stick infections in infants and decrease medical resource expenditure.

## 4. Discussion

### 4.1. Main Findings and Contribution

In this study, we demonstrated how machine learning assists clinical physicians in making clinical decisions regarding neonatal hypoglycemia outcomes. Our predictive models accurately forecasted neonatal hypoglycemia, providing practical value to healthcare practitioners. Importantly, we demonstrated that these models generalize well to future data. Hypoglycemia typically appears within the first few hours to days of birth. Neonates at high risk of hypoglycemia could be identified shortly after delivery and receive prophylactic interventions or additional monitoring for the clinical signs of disorder onset. Given that hypoglycemia typically occurs within a few hours after birth, our models enable the rapid identification of high-risk neonates. This facilitates the timely implementation of preventative interventions or further monitoring to detect early signs of disorder onset. Additionally, our models provided individual probabilities of neonatal hypoglycemia by decomposing the contribution of each risk factor, aiding physicians in understanding why a particular patient is classified as high-risk and enabling more targeted interventions to improve patient care.

The clinical validation analysis of AI assistance indicated that our best model can effectively lower medical expenses. It also implies a reduction in infection risk associated with frequent blood tests, alleviation of parental anxiety, and a lighter workload for medical staff, thereby lessening the healthcare system’s burden. Furthermore, we presented this AI web service application to physicians and nurses for evaluation and trial. They expressed high levels of acceptance towards the graphical interface and found the risk values to be reasonable, believing that it can significantly aid in clinical decision-making. Overall, the prediction model developed in this study holds academic value and has significant practical implications in clinical settings.

To our knowledge, this is one of the few studies that applied machine learning and big data techniques to practically predict neonatal hypoglycemia in well-baby nurseries. This study represents the first attempt to analyze the routinely available data on neonates admitted to well-baby nurseries at Chi Mei hospitals using AI/ML to predict the risk of developing early hypoglycemia. It stands as the most comprehensive study to date for predicting neonatal hypoglycemia.

### 4.2. Feature Importance Results and Clinical Implication

In traditional statistics, the use of Spearman rank order correlation methods and correlation coefficient matrices is a dependable statistical approach for examining the relationship between observed items. The correlation coefficient can range from −1 to 1, where −1 or 1 indicates a perfect relationship [45]. Our correlation results indicated that gestational age, mode of delivery, respiratory distress, birth weight < 2500 g, and maternal weight (BMI numerator) showed the strongest correlations with neonatal hypoglycemia (absolute value of correlation coefficient ranging from 0.118 to 0.245), while other feature variables exhibited relatively weak correlations with neonatal hypoglycemia.

In machine learning models, SHAP analysis was used to explain the contribution of each feature to the model. SHAP calculates the average difference between predicted values with and without the inclusion of each feature across all combinations, thereby elucidating the influence of inputs after machine learning [43,46]. This enabled the construction of a highly predictive model with good transparency for the outcomes. Our SHAP analysis on the RF model revealed that the top five features affecting the model quality are mode of delivery, gestational age, multiparity, respiratory distress, and birth weight < 2500 gm. Whether through correlation analysis or SHAP analysis, gestational age, mode of delivery, respiratory distress, and birth weight < 2500 gm emerge as the most critical influencing factors. This provides clinically significant insights for healthcare practitioners. Clinically, this suggests a need for enhanced glucose monitoring and early interventions for at-risk newborns, particularly those who are preterm, delivered by cesarean section, or experiencing respiratory distress.

### 4.3. Comparison with Related Studies

Table 4 demonstrates a comparison of the current study with other related studies predicting neonatal hypoglycemia using machine learning models [47,48,49]. The predictive model developed by Betts et al. [47] using XGBoost achieved the highest ROC curve value (0.82) among the studies; it also has the largest sample size. This model was based on routinely collected administrative data from the entire Australian state. However, it requires up to 532 feature variables, significantly increasing the data collection burden on clinical and professional coding staff in practical clinical use. Shukla et al. [48] used the Multiple Representations Sequence Miner (MrSQM) framework to predict neonatal hypoglycemia, achieving an ROC curve value similar to the present study. Their models were based on maternal continuous glucose monitoring (CGM) data. However, their study focused on infants born to mothers with diabetes, not all newborns. Gerard et al. [49] did not provide an AUC curve value, unlike the current and other related studies. They created predictive models using binary logistic regression to determine the need for oral dextrose glucose gel treatment in neonatal hypoglycemia, and were based on electronic health records (EHR) of maternal and neonatal data. Their study highlighted that the newborn’s first blood glucose value and maternal body mass index (BMI) are crucial variables in predicting the need for oral dextrose glucose gel treatment in neonatal hypoglycemia. Compared with other studies, our predictive model used fewer feature variables (12 variables) and achieved an AUC value of 0.735, effectively predicting neonatal hypoglycemia in all neonates in the well-baby nursery. Importantly, our AI models were implemented and integrated into the existing HIS supporting model usability.

In a recent meta-analysis examining the risk factors for neonatal hypoglycemia [50], researchers identified gestational age, birth weight, gestational diabetes, and maternal weight (BMI) as important predictors. This is consistent with the importance measure obtained in the study by Betts et al. [47] that gestational age, birth weight, and maternal diabetes arising during pregnancy were strong predictors. In the present study, the SHAP values were obtained for each individual for the strongest 12 predictors in the RF models for neonatal hypoglycemia.

### 4.4. Integrating the Model into the Existing Hospital Information System (HIS)

The significance of this study lies in the development of a prediction model for neonatal hypoglycemia using 12 readily available feature variables from electronic medical records, based on routinely collected administrative data. Most importantly, we established a predicted probability threshold, above which patients were classified as at risk of the outcome, and implemented the prediction model in a clinical setting. After confirming Random Forest as the best model, we integrated it with the existing HIS (nursery system and inpatient physician ordering system) to develop a web-based AI prediction application. Figure 4 displays a screenshot of the prediction application, showing a personalized prediction of the risk probabilities of hypoglycemia. Probabilities greater than or equal to 50% indicate a tendency for hypoglycemia to occur, while probabilities less than 50% suggest a lower likelihood of hypoglycemia. A higher probability indicates a greater risk. The implementation of this model in a clinical setting aids clinicians in the early identification of high-risk infants for neonatal hypoglycemia. As shown in Figure 4, the predicted probability is 79.6% showing a relatively high risk of hypoglycemia (i.e., AI predicted the baby would experience hypoglycemia within 4 h). This allows healthcare professionals to perform timely interventions such as breastfeeding, formula feeding, or administering glucose water, thereby preventing the onset of hypoglycemia. It also highlights the need for continuous blood glucose monitoring in newborns. Conversely, if the predicted risk probability of hypoglycemia is less than 50%, indicating a lower likelihood of occurrence, the newborn may avoid unnecessary repeated blood sampling.

### 4.5. Limitations and Future Directions

Some limitations in the current study have been observed. First, the findings indicated that the predictive model can only predict the risk of early neonatal hypoglycemia within the first four hours after birth. High-risk neonates may develop hypoglycemia beyond this period. Second, we collected our data from a single medical center in Southern Taiwan. The study population is not sufficiently large. Therefore, our model may not be generalizable to other populations or regions. Third, while our model demonstrated clinical validation with a predictive accuracy of 70.4% and a sensitivity of 100%, the specificity was 68.8%. This indicates that the model correctly identified 68.8% of newborns who did not have hypoglycemia, but 31.2% were falsely identified as at risk.

To address these issues, we plan to undertake the following in our future work. First, we will consider expanding the feature variables to include more detailed maternal health behaviors, nutritional status, and lifestyle data to improve the model’s accuracy and predictive capability. Second, multicenter data collection should be undertaken, covering different regions and multiple healthcare institutions, to validate the model’s applicability and generalizability in diverse clinical settings. Third, we plan to develop dynamic prediction models that can update in real-time according to changes in the newborn’s health status, such as blood glucose levels at different time points, which will enhance early intervention effectiveness. Finally, an external validation using real-world data should be conducted to continuously evaluate and refine the model, ensuring its reliability and practicality across diverse clinical settings.

## 5. Conclusions

This study is among the few that developed a neonatal hypoglycemia prediction model using 12 easily accessible feature variables from electronic medical records, derived from routinely collected administrative data, demonstrating the potential of such data in medical prediction. Integrating the prediction model with existing HIS and conducting real-world clinical testing is uncommon in the prevailing literature, highlighting the innovation of this study. By establishing a predicted probability threshold, the use of an AI application to provide personalized hypoglycemia risk predictions is an innovative approach that enhances the precision of clinical decision-making, significantly impacting neonatal care.

Overall, this study makes significant contributions to both academia and clinical practice. Our prediction model can assist clinicians in promptly predicting the risk of neonatal hypoglycemia shortly after birth, allowing for timely treatment. Additionally, the implementation of our model could help prevent hypoglycemia, reduce unnecessary blood glucose tests, lower infection risks, alleviate parental anxiety, improve care efficiency, and ultimately reduce healthcare costs.

## Figures and Tables

**Table 1:** The demographic and significant variable of early neonatal hypoglycemia (training dataset).

Variables	Euglycemia (*n* = 1374, 73.1%)	Hypoglycemia (*n* = 506, 26.9%)	*p*-Value
Sex, *n* (%)			0.372
male	718 (52.26)	252 (49.80)	
female	656 (47.74)	254 (50.20)	
Gestational age, mean (SD)	38.64 (1.33)	37.82 (1.56)	<0.001
BW < 2500 gm, *n* (%)	326 (23.73)	191 (37.75)	<0.001
BW > 4000 gm, *n* (%)	66 (4.80)	30 (5.93)	0.387
Mode of delivery, *n* (%)			<0.001
Vaginal delivery	825 (60.04)	193 (38.14)	
Cesarean section	549 (39.96)	313 (61.86)	
Head circumference, mean (SD)	33.30 (1.77)	33.27 (1.84)	0.826
Chest circumference, mean (SD)	31.45 (2.27)	31.36 (2.73)	0.514
Birth length, mean (SD)	49.19 (2.91)	48.91 (3.32)	0.102
Apgar score 1 min, mean (SD)	7.95 (0.29)	7.89 (0.43)	0.005
Apgar score 5 min, mean (SD)	8.99 (0.13)	8.97 (0.18)	0.065
Clinical sepsis, *n* (%)	49 (3.57)	33 (6.52)	0.008
Respiratory distress, *n* (%)	219 (15.94)	157 (31.03)	<0.001
Polycythemia, *n* (%)	13 (0.95)	17 (3.36)	<0.001
Body temperature, mean (SD)	36.55 (0.58)	36.40 (0.64)	<0.001
Maternal age, mean (SD)	32.12 (4.78)	32.45 (4.74)	0.181
Maternal weight, mean (SD)	67.48 (11.55)	69.88 (11.92)	<0.001
Maternal height, mean (SD)	159.30 (5.44)	159.96 (5.69)	0.025
Multiparity, mean (SD)	1.54 (0.79)	1.60 (0.76)	0.090
Prior delivery of SGA, *n* (%)	93 (6.77)	29 (5.73)	0.481
Prior delivery of LGA, *n* (%)	51 (3.71)	24 (4.74)	0.379
Gestational diabetes, *n* (%)	186 (13.54)	87 (17.19)	0.055
Preeclampsia, *n* (%)	49 (3.57)	29 (5.73)	0.050
HBsAg (+), *n* (%)	85 (6.19)	34 (6.72)	0.753
PROM > 24 h, *n* (%)	24 (1.75)	15 (2.96)	0.144

PROM: Premature rupture of membrane. Continuous variables were reported as the mean and standard deviation (SD). Categorical variables were presented as frequency counts with percentages. Variables were evaluated using Student’s *t*-test for continuous variables and Pearson’s chi-squared test for categorical variables. *p* Value of <0.05 was considered statistically significant.

**Table 2:** Spearman’s correlation coefficients between features and early neonatal hypoglycemia.

Variables	Correlation Coefficients
Gestational age	−0.2449
Mode of delivery	0.2133
Respiratory distress	0.1711
BW-2500	0.1403
Maternal weight	0.1178
Body temperature	−0.1144
Apgar score 1 min	−0.1070
Polycythemia	0.0833
Apgar score 5 min	−0.0707
Gestational diabetes	0.0678
Maternal height	0.0677
Multiparity	0.0650
BW-4000	0.0573
Maternal age	0.0539
Preeclampsia	0.0506
Sex	−0.0489
Prior delivery of LGA	0.0456
Clinical sepsis	0.0325
Prior delivery of SGA	−0.0321
Birth length	−0.0309
PROM 24 h	0.0295
Chest circumference	−0.0292
Head circumference	0.0185
HBSAg (+)	0.0065

**Table 3:** Model performance with 12 features for prediction of early neonatal hypoglycemia.

Predictive Models	Accuracy	Sensitivity	Specificity	F1 Score	Precision	AUC
Stacking	0.689	0.682	0.692	0.541	0.448	0.739
Random Forest	0.658	0.682	0.649	0.517	0.417	0.732
Voting	0.675	0.682	0.673	0.530	0.434	0.732
AdaBoost	0.646	0.682	0.632	0.509	0.405	0.723
XGBoost	0.647	0.691	0.631	0.513	0.408	0.722
Logistic Regression	0.675	0.687	0.671	0.532	0.434	0.721
MLP	0.675	0.682	0.673	0.530	0.434	0.721
LightGBM	0.646	0.682	0.632	0.509	0.405	0.717
SVM	0.656	0.650	0.658	0.504	0.411	0.713

**Table 4:** A comparison with related studies.

Study	This Study	Betts et al. [47]	Shukla et al. [48]	Gerard. et al. [49]
Study design and setting	Retrospective study Routine administrative data on neonates born ≥35 weeks of gestational age.	Retrospective study Routine administrative data on neonates born <39 weeks of gestational age.	Retrospective study Maternal continuous glucose monitoring (CGM) data for neonates born to mothers with diabetes.	Retrospective study Electronic health record (EHR) neonates
Sample size	2687	154,755	90	13,476
machine learning algorithms	Logistic Regression, Random Forest, Light GBM, XG Boost, MLP	Gradient boosted trees, Logistic regression	Multiple Representations Sequence Miner (MrSQM) framework	Logistic regression
Feature variables	The 13 variables consist of maternal and neonatal clinical data routinely collected and recorded immediately after birth.	The 528 variables include all available maternal clinical, demographic, and lifestyle data, as well as neonatal clinical data routinely collected and recorded immediately after birth	**1 variable** maternal continuous glucose monitoring (CGM) data	Maternal data (All acute and chronic diagnoses for maternal patients and diagnosed issues in newborn patients) and neonatal data (all conditions billed for during care)
outcome	early neonatal hypoglycemia	neonatal hypoglycemia	neonatal hypoglycemia,	neonatal hypoglycemia
Testing results	AUC of 0.735	AUC = 0.832	AUC of 0.74	*p* < 0.001
Best predicting model	Random Forest	Gradient boosted trees	Multiple Representations Sequence Miner (MrSQM) framework	binary logistic regression model
Real world implementation	Yes	None	None	None

## Data Availability

The dataset used for this study is available upon request from the corresponding author.

## Appendix A

**Table A1:** Hyper-parameters range for experiments.

Algorithm	Hyper-Parameters
Logistic Regression (LR)	LR__C: [0.001, 0.01, 0.1, 1, 10, 100] LR__max_iter: [100, 200, 300, 400, 500, 1000] LR__penalty: [l1, l2] LR__class_weight: [‘balanced’, None] LR__solver: [‘lbfgs’, ‘saga’, ‘liblinear’]
Random Forest (RF)	n_estimators: [100, 200, 300, 400, 500] max_depth: [None, 4, 5, 6, 8, 10, 20, 30] class_weight: [‘balanced’, {0: 1, 1: 3}] min_samples_split: [6, 8, 10, 12, 15, 20, 30] min_samples_leaf: [5, 6, 8, 10, 15] max_features: [‘auto’, ‘sqrt’, ‘log2’]
Support Vector Machine (SVM)	SVM__kernel: [rbf, linear] SVM__gamma: [1e-3, 1e-4] SVM__C: [1, 10, 100, 500] shrinking: True, False
LightGBM	learning_rate: [0.001, 0.01, 0.1, 1] n_estimators: [100, 200, 300, 400, 500] max_depth: [4, 5, 10, 12, 15, 20, 30] random_state: [8, 16, 42, 57, 66] class_weight: [‘balanced’] importance_type: [‘gain’]
MLP (Multi-layer Perceptron)	hidden_layer_sizes: [(50, 30)] learning_rate_init: [1e-3, 1e-2, 1e-1] max_iter: [200, 100, 50] early_stopping: [True, False] alpha: [1e-3, 1e-4, 1e-5]
XGBoost	n_estimators: [100, 200, 300, 400, 500] learning_rate: [0.001, 0.01, 0.05, 0.1] gamma: [0, 5, 10] scale_pos_weight: [1, 2, 3]
Voting	Voting: [hard, soft]
Stacking	final_estimator: [Logistic regression]
AdaBoost	base_estimator: [DecisionTreeClassifier] max_depth: [1, 2, 3, 5] n_estimators: [50, 100, 200, 400] learning_rate: [0.001, 0.01, 0.1, 1.0]

Note. The hyper-parameters that are not described in this table are set to the default values used in the scikit-learn library.
